# Temporal bias in case-control design: preventing reliable predictions of the future

**DOI:** 10.1038/s41467-021-21390-2

**Published:** 2021-02-17

**Authors:** William Yuan, Brett K. Beaulieu-Jones, Kun-Hsing Yu, Scott L. Lipnick, Nathan Palmer, Joseph Loscalzo, Tianxi Cai, Isaac S. Kohane

**Affiliations:** 1 Department of Biomedical Informatics, Harvard Medical School, Boston, MA USA; 2 Department of Stem Cell and Regenerative Biology, Harvard University, Cambridge, MA USA; 3 Center for Assessment Technology and Continuous Health, Massachusetts General Hospital, Boston, MA USA; 4 Department of Medicine, Brigham and Women’s Hospital, Boston, MA USA; 5 Division of Data Sciences, VA Boston Healthcare System, Boston, MA USA; 6 Department of Biostatistics, Harvard T. H. Chan School of Public Health, Boston, MA USA

**Keywords:** Machine learning, Epidemiology

## Abstract

One of the primary tools that researchers use to predict risk is the case-control study. We identify a flaw, temporal bias, that is specific to and uniquely associated with these studies that occurs when the study period is not representative of the data that clinicians have during the diagnostic process. Temporal bias acts to undermine the validity of predictions by over-emphasizing features close to the outcome of interest. We examine the impact of temporal bias across the medical literature, and highlight examples of exaggerated effect sizes, false-negative predictions, and replication failure. Given the ubiquity and practical advantages of case-control studies, we discuss strategies for estimating the influence of and preventing temporal bias where it exists.

## Abstract (web summary)

One of the primary tools that researchers use to predict risk is the case-control study. Here, the authors identify a flaw, temporal bias, that is specific to and uniquely associated with these studies that occurs when the study period is not representative of the data that clinicians have during the diagnostic process, undermining the validity of predictions.

## Introduction

The ability to predict disease risk is a foundational aspect of medicine, and is instrumental for early intervention, clinician decision support, and improving patient outcomes. One of the main tools utilized by researchers for identifying predictive associations or constructing models from observational data is the case-control study^1^. By measuring differing exposure patterns between the case and control groups, exposures can be interpreted as predictors or risk factors for case status^2,3^. With the proliferation of observational datasets and novel machine learning techniques, the potential for these studies to play a direct role in personalized medicine has begun to be explored^4^. However, we have identified a structural flaw, seen widely in basic case-control study designs, which we call temporal bias. At its core, temporal bias represents a mismatch between the data used in the study and the data that a clinician would have access to when making a diagnostic decision. A clinician must evaluate all patients in real time, without the luxury of knowing that they have been pre-selected according to their future status. Case-control studies, as popularly implemented, are uniquely unable to make prospectively valid predictions. This temporal bias not only amplifies reported effect sizes relative to what would be observed in practice, but also obfuscates the prospective use of findings.

A classic example of temporal bias and its impacts can be seen through the initial discovery of lyme disease, a tick-borne bacterial infection. Lyme disease is characterized by (i) an initial bite, (ii) an expanding ring rash, and (iii) arthritic symptoms, in that order^5^. However, the original 1976 discovery of lyme disease (then termed lyme arthritis) focused exclusively on patients who manifested with arthritic symptoms^6^. This enabled researchers to definitively identify the prognostic value of a ring rash towards arthritis, but not tick bites, due to the latter symptom’s temporal distance from the researcher’s focus. By focusing on predictive features immediately prior to the event in question, researchers capture a biased representation of the full trajectory from healthy-to-diseased. A contemporaneous doctor aware of lyme arthritis examining a patient presenting with a tick bite would miss the possibility of disease until further symptoms developed. Similarly, a predictive model for lyme arthritis focused on ring rashes would report false negatives if it were deployed in practice: patients who had yet to develop ring rashes would contract arthritis at a future time. These errors stem from the incomplete picture of symptoms that was captured.

However, temporal bias is not a problem of the past. The central flaw, an overemphasis on features collected near the case event, still occurs in the literature today. Within the medical domain, there are numerous examples of temporal bias in both clinical medicine and machine learning^7–16^. Despite increasing interest in machine learning risk prediction, few tools for use on individual patients have become standard practice^17,18^. As algorithms trained using large datasets and advanced machine learning methods become more popular, understanding limitations in the way they were generated is critical. In this article, we describe the basis for temporal bias and examine three representative instances of temporal bias in the medical, machine learning, and nutritional literature to identify the impact that this phenomenon has on effect sizes and predictive power.

Of interest are the expansive set of studies that focus on predicting future events in real time and obey the following general conditions. First, events to be predicted take the form of state transitions (healthy-to-diseased, stable-to-failed, control-to-case, etc.). This implies that there exists a bulk population of controls, from which cases differentiate themselves. Soon-to-be cases progress along a trajectory away from the control population at varying speeds. This trajectory terminates at the occurrence of the case event, but the position of control individuals along this trajectory cannot be reliably determined.

Second, we consider that the risk-of-event is equivalent to measuring progress along a control-to-case trajectory in time. Because risk prediction utilizes features from the present to assess the chance of a future event occurring, an event that is truly random would not be appropriate for a risk prediction algorithm. The trajectory represents the ground truth progression along a pathway towards the event in question and are defined relative to the specific populations chosen for the study. This assumes that the researchers have taken the exchangeability^19^ of their case and control populations into account: if members of the control population are chosen poorly and cannot experience the case event, then there can be no trajectory.

Third, at the population level, the trajectory commences when the to-be-diseased population first begins to diverge from the non-diseased population and reaches a maximum when the disease event actually occurs. This requires that the trajectory is aligned to the event in question. Diseased individuals must consequently be referred to using terms such as days to disease, while control individuals exist in an undefined point along this timeline, because their days to disease is unknown. This is only required due to the retrospective nature of these studies and is a major departure from prospective deployment.

Finally, the features actually measured by a study represent proxies for an individual’s position along the trajectory. Regardless of their positive or negative association with the event, features subject to temporal bias will tend to diverge between cases and controls with a continuous trajectory, and become better at differentiating the controls from cases as case individuals get closer to their event. This divergence provides the mechanism of action for temporal bias to act. If a model does not possess time varying features (such as a GWAS), temporal bias cannot occur, but predicted risk will also be static with respect to time-to-case-event.

**Fig. 1:**
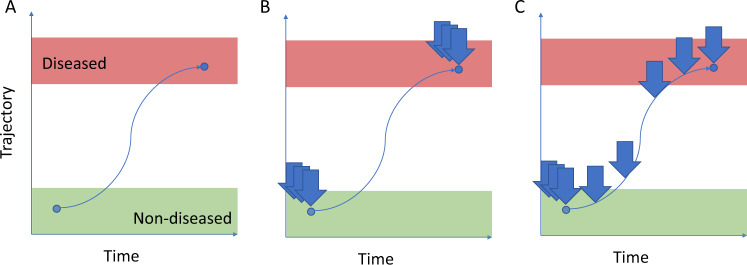
Prospective risk can be represented as a trajectory. Red and green zones represent positions on the trajectory corresponding to outward definitions of diseased and non-diseased status. Vertical arrows represent sampling of population at a particular point of a trajectory. **A** The (single-class) case-control paradigm often imposes a dichotomous (binary) framework onto a continuous trajectory. **B** Experiments utilizing observations of cases that are concentrated at the time when the case event occurs cannot capture any information regarding the transition trajectory, resulting in temporal bias. **C** In order to predict a patient’s position along the trajectory, experiments capturing the entire transition from non-diseased to diseased are necessary.

As a result, we can distill prediction studies into a common structure (Fig. 1): the members of the diseased population begin as controls at a point in the past, and progress along a trajectory until the disease occurs. Most case-control studies apply a dichotomous framework over this continuous trajectory.

Temporal bias occurs when cases are sampled unevenly in time across this trajectory (Fig. 1B). (A theoretical basis for temporal bias is presented in Supplementary Note 1.) This is a separate but analogous effect compared to selection bias: the control population may be exchangeable with the diseased population but must tautologically exist at a prior point along the disease trajectory compared to cases. Rather than operating over the selection of which patients to include in the study, temporal bias acts over the selection of when each subject is observed.

This important temporal feature yields two implications:

1. If the features of diseased subjects are evaluated based on a point or window that is defined relative to the case event (a future event, from the perspective of the feature measurements), features in the end of the trajectory will be oversampled. For example, a study that compares individuals one year prior to disease diagnosis to healthy controls will oversample the trajectory one year prior to disease, and undersample the trajectory further out.
2. The resulting model cannot be prospectively applied because the study design implicitly leaked information from the future: a prospective evaluator has no way of knowing if a particular subject is within the observation window defined by the study. It cannot be known if an individual is one year away from a disease diagnosis in real time.

Temporal bias is intuitively understood within certain epidemiological circles- in fact:

recall bias, caused by the tendency for survey respondents to remember recent events at a higher rate relative to past events, can be interpreted as a specific instance of temporal bias. Similarly, it is understood that case-control studies represent a lower level of evidence relative to other study designs^20^. Methodologies have been proposed that, while not explicitly designed to address temporal bias, happen to be immune to it (density-based sampling, among others^21^). However, these tend to focus on point exposures or necessitate impractically exact sampling strategies. Despite this important shortcoming, the ease of the case-control framework has allowed temporal bias to proliferate across many fields. We examine three examples, in cardiology, medical machine learning, and nutrition below.

## Results

### Temporal bias can inflate observed associations and effect sizes

The INTERHEART study^22^ examined the association between various risk factors and myocardial infarction (MI) using a matched case-control design among a global cohort. Individuals presenting at hospitals with characteristic MI were defined as cases, and subjected to interviews and blood tests, while matched controls were identified from relatives of MI patients or healthy cardiovascular individuals presenting with unrelated disorders. One risk factor of interest included lipoprotein (a) [Lp(a)], a blood protein^23,24^. While Lp(a) levels are thought to be influenced by inheritance, significant intra-individual biological variance with time has been reported^25,26^.

One recent analysis utilized data from this study to examine the positive association between blood levels of Lp(a) and MI across different ethnicities and evaluate the possible efficacy of Lp(a) as a risk prediction feature^27^. However, because cases were only sampled at the time of the MI event, the resulting effect sizes are difficult to interpret prospectively. Indexing case patients by their case status leaks information regarding their status to which a physician prospectively examining a patient would not have access to. Intuitively, if Lp(a) was static until a spike immediately prior to an MI event, it could not be used as a prospective risk predictor, even though a significant association would be observed given this experimental design. This limitation cannot be overcome using only the data that was collected, as information regarding the dynamics of Lp(a) over time is missing. To evaluate the influence of temporal bias, we estimated the size of the Lp(a)-MI association had the experiment been done prospectively. This analysis was done by simulating control-to-case trajectories using INTERHEART case/control population Lp(a) distributions by imputing the missing data. We conducted extensive sensitivity testing over different possible trajectories to evaluate the range of possible effect sizes. This approach allowed for the recalculation of the association strength as if the study had been conducted in a prospective manner from the beginning.

Table 1 summarizes the observed effect size in the simulated prospective trials compared to the reported baseline. In all cases, the simulated raw odds ratio between Lp(a) and MI was significantly lower than the observed raw odds ratio due to temporal bias present in the latter measurement. This is intuitive, since case individuals as a group will be more similar to controls (healthier) when sampled at random points in time rather than when they experience an MI event (Fig. 1B). Although it cannot be proven that prospective effect sizes would be smaller, as this would require longitudinal data that do not exist, this experiment suggests that the degree of temporal bias scales with area under the imputed trajectory. In order to observe the reported odds ratio, the underlying trajectory would need to resemble a Heaviside step function in which cases spontaneously experience a spike in Lp(a) levels at the point of their divergence from the controls, an assumption that is neither explicitly made in the study nor has a basis in biology. We repeated the imputation process with Heaviside step function-based trajectories, varying the position of the impulse in the trajectory (Table 1). As the impulse location approaches the beginning of the trajectory, the effect size relative to the baseline approaches 1. This observation illustrates the assumption intrinsic in the original INTERHEART experimental design: that MI individuals had static Lp(a) measurements during the runup to their hospitalizations.

**Table 1:** The observed Lp(A)-MI association is magnified by temporal bias.

Initial case state imputation method	Trajectory type	Effect size relative to reported baseline (95% CI)
Weighted sampling	Linear	0.172 (0.160–0.196)
Weighted sampling	Logistic	0.169 (0.150–0.187)
Weighted sampling	Logarithmic	0.403 (0.390–0.417)
Percentile matching	Linear	0.518 (0.507–0.528)
Percentile matching	Logistic	0.517 (0.506–0.527)
Percentile matching	Logarithmic	0.639 (0.631–0.647)
Percent shift	Linear	0.389 (0.376–0.401)
Percent shift	Logistic	0.386 (0.373–0.399)
Percent shift	Logarithmic	0.539 (0.530–0.549)
Weighted sampling	HSF: Impulse in first 10% of trajectory	0.808 (0.801–0.814)
Weighted sampling	HSF: Impulse in first 1% of trajectory	0.980 (0.977–0.984)
Weighted sampling	HSF: Impulse in first 0.1% of trajectory	0.998 (0.997–0.999)
Percentile matching	HSF: Impulse in first 10% of trajectory	0.898 (0.895–0.901)
Percentile matching	HSF: Impulse in first 1% of trajectory	0.989 (0.989–0.989)
Percentile matching	HSF: Impulse in first 0.1% of trajectory	0.999 (0.999–0.999)
Percent shift	HSF: Impulse in first 10% of trajectory	0.860 (0.857–0.864)
Percent shift	HSF: Impulse in first 1% of trajectory	0.987 (0.985–0.989)
Percent shift	HSF: Impulse in first 0.1% of trajectory	0.999 (0.999–0.999)

Association effect sizes from 100 simulated prospective trials each relative to INTERHEART sizes. Effect sizes less than 1 represent smaller simulated effects compared to those from INTERHEART.

*HSF* Heaviside step function.

To characterize these findings in a real-world dataset, we examined the Lp(a) test values and MI status of 7128 patients seen at hospitals and clinics within the Partners Healthcare System-representing Brigham and Women’s Hospital and Massachusetts General Hospital among others-who had indications of more than one Lp(a) reading over observed records. This dataset included 28,313 individual Lp(a) tests and 2587 individuals with indications of myocardial infarction. We identified significant intra-individual variation in Lp(a) values in this population: the mean intra-individual standard deviation between tests was 12.2 mg/dl, compared to a mean test result of 49.4 mg/dl. These results are consistent with literature findings of significant intra-individual variance of Lp(a) values^25,28,29^, challenging the assumption that individuals could have static levels in the runup to MI. Furthermore, in this dataset, biased Lp(a) measurement selection among case exposure values varied the observed association strength between Lp(a) and MI by between 51.9% (preferential selection of lower values) to 137% (preferential selection of higher values) of what would have been observed with random timepoint selection. On the upper end, this is a conservative estimate: we would expect the deviation to increase upon correcting for ascertainment bias in the dataset. Control individuals would be less healthy than true controls, while cases would typically not be sampled immediately prior to an MI, and consequently appear to be healthier than INTERHEART cases. These findings suggest that temporal bias was likely to act in this study design as executed, in a manner that would reduce the observed utility of Lp(a) as a risk predictor for future MI.

### Prospective prediction failure due to temporal bias

As the availability of observational data has skyrocketed, event prediction has become a popular task in machine learning. Because of this focus on prediction, many methods utilize the idea of a prediction window: a gap between when an event is observed and when features are collected^12,13^. A model that differentiates patients six months prior to MI onset from healthy matched controls may be said to detect MI six months in advance. However, because the window is defined relative to a case event, it represents an uneven sampling of the disease trajectory. Consequently, this prediction requires unfounded assumptions regarding the trajectory of MI onset. For example, if the trajectory is such that patients’ risk in the year prior to the MI is approximately uniform and significantly elevated from the control risk, a model trained in this way would provide many false positive 6-month MI predictions by falsely implicating patients more than 6 months away from an MI. Because window sizes are often chosen without respect to the underlying transition trajectory, significant potential for temporal bias still exists, driven by factors such as differential diagnosis periods or missed exposures.

To illustrate the impact of temporal bias in this case, we constructed predictors for childbirth: a phenotype that was chosen because of its well-defined trajectory. While the trajectory for delivery is a rare example of a step function, we demonstrated in the previous section that the use of case-control effectively imposes a step-function regardless of the true shape of the underlying trajectory. Rather than to present a toy example, this is intended to represent the extreme case of the potential consequences of releasing a predictive model trained in this manner.

**Fig. 2:**
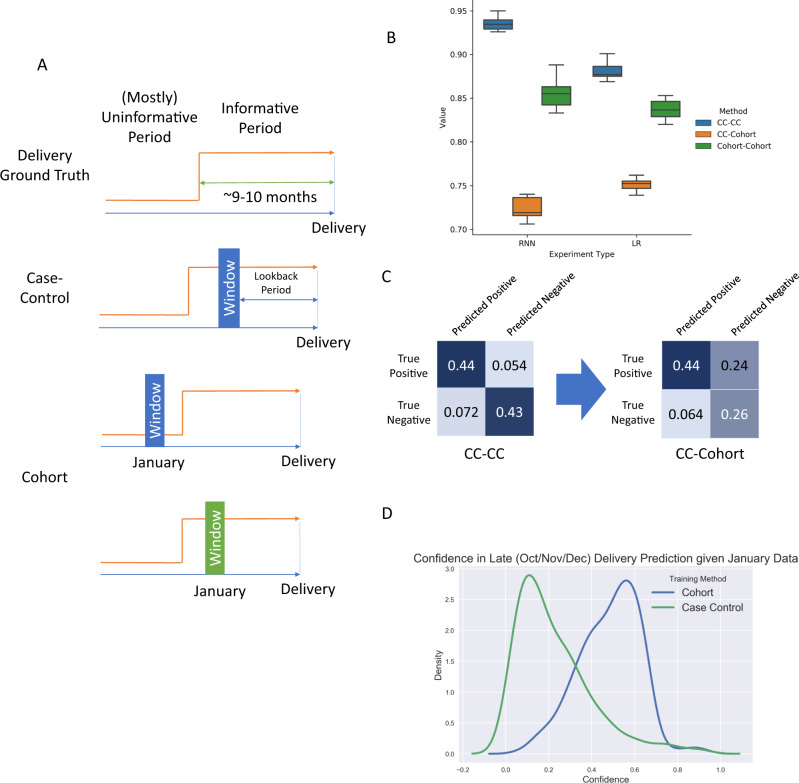
Case-control predictors for delivery report false negatives when applied prospectively due to temporal bias. **A** The ground truth trajectory for delivery (orange) is composed of parts: an informative period, 9–10 months prior to the delivery, and a largely uninformative period prior. Case-control windows (blue) are indexed to delivery/baseline date, and so only sample a single (informative) slice of the trajectory. Cohort windows (green) always occur in January, and so uniformly sample the trajectory. **B** Model performance (Validation AUROC) for deep recurrent neural networks and logistic regression for each study design. Error bars represent the 95% confidence intervals. Each box represents the results of 10 independently trained models. Box bounds represent upper quartile, lower quartile, and mean. Whiskers represent maxima and minima. **C** Comparison of confusion matrices for CC-CC (left) and CC-Cohort (right) models. Color intensity corresponds to matrix value. **D** CC-Cohort validation model confidence distributions for late (Oct/Nov/Dec) deliveries given January features.

In this system, cases and controls are significantly more difficult to distinguish more than nine or ten months prior to delivery compared to later in pregnancy because the case population is not yet pregnant. Features collected while the case population is pregnant are far more informative regarding delivery status. A case-control study that uses a window defined three months prior to delivery will capture these informative, pregnancy related features. In contrast, a cohort study examining all patients in January of a given year will capture largely uninformative features when the case individual’s delivery takes place late in the year (Fig. 2A).

Using 2015 data from a de-identified nationwide medical insurance claims dataset, we simulated three studies:

I. CC-CC: models trained and evaluated under the case-control (CC) paradigm: one month of records, three months prior to the delivery date (cases) or matched baseline date (controls) are used.
II. CC-Cohort: models trained under the case-control paradigm, but evaluated under the cohort paradigm, where records from January are used to predict delivery in 2015.
III. Cohort-Cohort: models trained and evaluated under the cohort paradigm.

For each simulated study, records within the observation window of diagnoses, procedures, and prescriptions ordered were fed into both deep recurrent neural nets (RNN) and logistic regression (LR) models.

The significant difference in performance (Fig. 2B) between CC-CC and CC-Cohort models illustrates a central trait of temporally-biased sampling. Uneven sampling across the transition trajectory improves validation AUC under artificial validation conditions, but model performance collapses when deployed in a prospective manner. In contrast, models designed with the prospective task from the outset (Cohort-Cohort) had intermediate performance that reflected the inherent ambiguity of the available observations. These findings were robust across both RNN and LR-based models. In fact, while the more complex RNN performed better than the logistic regression model for the CC-CC task, it performed worse than the LR on the CC-Cohort task. In this case, methodological improvements on an unrealistic task led to more significant declines in performance on a more realistic task.

For women with October/November/December deliveries, claims data from January are mostly uninformative, and a reliable prediction at that point is not possible at the population level, especially when using features trained during pregnancy. The confusion matrices produced by CC-CC and CC-Cohort models revealed that much of the performance collapse can be traced to false negatives (Fig. 2C). We examined the confidence that the deep convolutional networks assigned to October/November/December deliveries when evaluated on cohort structured data were predictive (Fig. 2D). Models trained under using case-control incorrectly label these individuals as high confidence controls, while models trained using cohorts more appropriately capture the intrinsic ambiguity of the prediction task. Clinicians do not have the luxury of examining only patients three months/six months/one year prior to disease incidence: they must assess risk in real time. These studies are common in the machine learning literature- one study even described the act of aligning patients by disease diagnosis time as a feature, and a major reason why their framework was better able to stratify risk^14^. However, aligning patients in this way requires waiting until disease diagnosis, and so the superior risk stratification comes too late to be useful.

It is critical to note that this is a problem that cannot be solved methodologically. As evidenced by the comparison of the performance of the RNN and LR models, novel or exotic machine learning techniques cannot compensate for the fact that the data fed into the models represent a distorted view of the actual population distribution that would be encountered prospectively. Even with perfect measurement and modeling, temporal bias and the issues that result would still be present: the underlying trajectory would still be unobserved.

### Temporal bias-induced replication failure

**Fig. 3:**
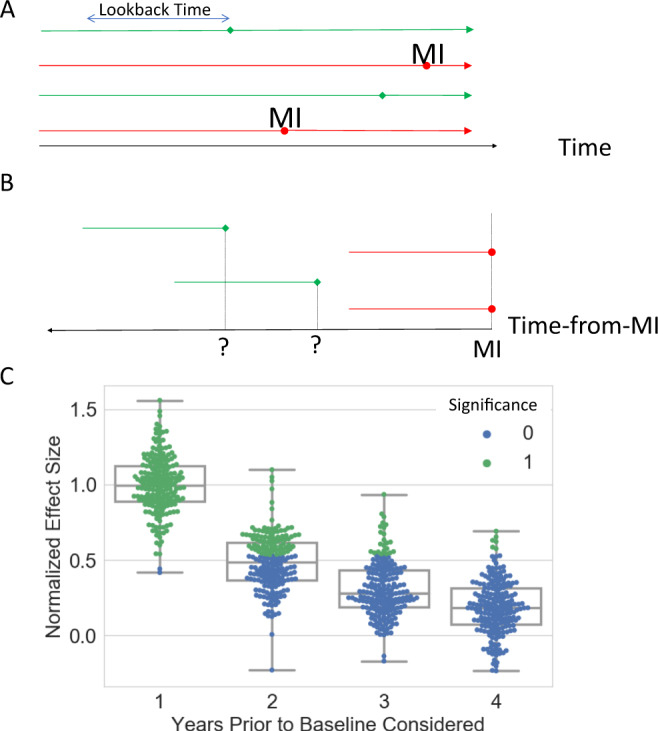
Temporal bias results from arbitrary alignment of cases and future-indexed lookback times. **A** Over a particular time period, longitudinal data of olive oil consumption is continuous for all cohort members with time. Circles represent MI events, while diamonds represent matched, but otherwise arbitrarily chosen baseline points for controls. **B** Case-control studies arbitrarily align MI patients at the date of the MI. As a result, the time dimension is inverted and anchored to the MI date, the position of controls is consequently lost. **C** Strength of olive oil consumption-MI association given years of consumption prior to baseline considered. Effect size is normalized to the average 1-year association strength. Points are colored based on statistical significance after FDR correction. Each box plot represents 200 repeated trials. Box bounds represent upper quartile, lower quartile, and mean. Whiskers represent maxima and minima.

Studies that identify disease risk factors through nutrition data enjoy a particularly high profile among the public^30^. As an example, the Mediterranean diet (characterized by consumption of olive oil, fruits, vegetables, among other factors) has been implicated as a protective factor against coronary heart disease, but the mechanism for this association is unclear. One paper set out to examine whether olive oil consumption specifically was associated with MI using patients from a Spanish hospital^31^. MI patients and matched controls were interviewed regarding their olive oil consumption over the past year, and a protective effect against MI was observed among the highest quintile of olive oil consumers. In response, another group analyzed data from an Italian case-control study and were unable to identify the same association between the upper quintile of olive oil consumption and MI^32^. Crucially, these analyses differed in the size of the observation window used: one year and two years respectively. As a result, not only were these studies sampling the MI trajectory unevenly, they sampled different parts of the MI trajectory. To examine the degree to which differing amounts of temporal bias present in each study could have influenced the results of the study, we utilized longitudinal data from nearly 100,000 individuals from the Nurses’ Health Study (NHS) regarding olive oil consumption patterns and MI to provide a baseline ground truth. We simulated retrospective case-control studies that considered different lookback periods to determine if the presence or magnitude of a protective effect was sensitive to the manner in which an experiment was conducted. Figure 3A details the simulation setup: longitudinal records (Fig. 3A) were used to identify case (red) and control (green) individuals. MI dates were identified for cases, and baseline dates for controls were selected to match the age distribution of the cases. For each patient, exposures during the lookback time are recorded. The association between MI and the observed exposures were then calculated and the influence of the lookback time on association strength was assessed.

The simulated studies that examined one year of past olive oil consumption relative to the MI/baseline date detected a protective effect, as originally observed. However, the magnitude and statistical significance of this effect decayed as the size of the lookback period was increased, consistent with the results of the failed replication. When a two-year lookback period was used, only 41% of simulated studies observed a statistically significant result (Fig. 3C). The observed protective effect in these cases is an artifact of methodology, rather than medicine, physiology, or society. The act of looking back from the MI date/matched baseline has the effect of inverting the time axis to time-from-MI “and aligning the case individuals (Fig. 3B). However, no such treatment is possible for control individuals, and their position along the new temporal axis is unknown. As a result, there is no functional basis for comparing healthy individuals to individuals artificially indexed to a future event (MI) because these represent groups that can only be identified retrospectively, after the MI has already occurred. A mismatch exists between the information utilized in the study and the information that patients or physicians would have access to when making dietary decisions. While there may indeed be a prospective association between olive oil and MI, protective or otherwise, the data to observe such an effect was not collected. Because both olive oil consumption and MI risk are time-varying features, the strength of the instantaneous association between the two will naturally depend on when each feature is measured.

## Discussion

Temporal bias can be thought of as a flaw present in the application of case-control experiments to the real-world diagnostic or prognostic task. Because these experiments do not uniformly sample the control-to-case trajectory, features and observations in certain parts of the trajectory are oversampled and assigned disproportionate weight. These observations also do not match the observations that physicians or patients have when assessing risk in real time. Because the case observations that are model-applicable can only be identified after the case event actually occurs, the resulting experimental findings are impossible to use prospectively. Temporal bias serves to amplify differences between the healthy and diseased populations, improving apparent predictive accuracy and exaggerating effect sizes of predictors. In prospective cases, it may also result in researchers failing to discover predictive signals that were outside the window considered. Because the magnitude of its effects is a function of an often-unobserved trajectory, temporal bias is poorly controlled for and can lead to replication bias between studies. The relative impact of temporal bias will scale with the dynamic range of the trajectory: a trajectory that contains large, dramatic changes is susceptible to bias, while trajectories composed of static features (genotype, demographics, etc) will largely be immune.

Temporal bias has existed alongside case-control studies from when they were first utilized. The first documented case-control study in the medical literature was Reverend Henry Whitehead’s follow-up^33^ to John Snow’s famous report^34^ on the Broad Street cholera outbreak. Whitehead aimed to evaluate Snow’s hypothesis that consuming water from the Broad Street pump led to infection. Whitehead surveyed both families of infected and deceased as well as individuals without cholera regarding their consumption of pump water during the time deaths were observed^35,36^.

**Fig. 4:**
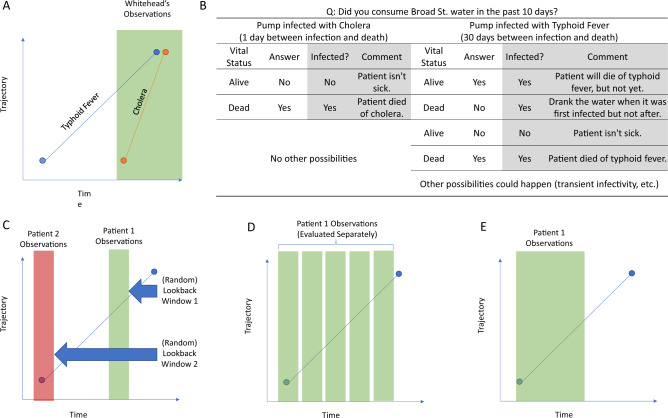
Preventing temporal bias. **A** Whitehead’s cholera study benefited from the short period between infection and death. Had Whitehead been faced with an outbreak of typhoid fever, his sampling strategy would oversample late-stage features. **B** Hypothetical interview data from Whitehead’s case-control study. Lacking underlying knowledge regarding disease etiology, Whitehead’s experimental design would have experienced temporal bias given a disease with a longer incubation period. Shaded columns represent information hidden to the investigator. **C** Randomizing the lookback window among case patients can uniformly sample the trajectory, if the lookback times go far back enough. **D** Evaluating person-days, person-weeks, or person-months can allow for the entire trajectory to be considered. **E** Conducting a cohort study by creating a well-defined date from which a look forward window is deployed does not uniformly sample the trajectory in all individuals, but is still prospectively implementable since the starting date can be determined in real time.

The outbreak began on August 31st, 1854^34^, with deaths occurring in the days that immediately followed. Whitehead’s efforts in identifying pump-water exposure among outbreak victims focused on the time period between August 30th and September 8th, corresponding to a lookback time between 1 and 10 days, depending on when the victim died. This would normally result in temporal bias towards the end of cholera trajectory. Although Whitehead’s conclusions were ultimately correct, the brief incubation period (2 h to 5 days^37^) of cholera contributed to the success of the experiment and Whitehead’s later ability to identify the index patient. The rapid transition from healthy to diseased ensured that Whitehead’s chosen lookback time would have uniformly sampled the disease trajectory but is also something Whitehead could not have known at the time. Had Whitehead instead been faced with an outbreak of another waterborne disease such as typhoid fever, which can have an incubation period as long as 30 days^38^, Whitehead’s chosen window would oversample exposure status in the runup to death, leading to temporal bias that would overemphasize features in the latter portion of the disease trajectory (Fig. 4A). Because the disease etiology and trajectory were unknown at the time, the association between Broad Street water and death is much less clear in the case of a hypothetical typhoid fever epidemic. (In another instance with unclear etiology, a recent survey of COVID-19 predictive algorithms found a significant number utilizing case-control sampling^39^). Figure 4B summarizes hypothetical interview data given Whitehead’s study design in the case of both a cholera and a typhoid fever outbreak. In the unshaded columns, which represent information he would have access to, the association between pump water consumption and mortality is only clear in the case of cholera.

Many factors have contributed to unconscious adoption of bias-susceptible experimental designs. From a data efficiency perspective, case-control studies are often motivated by large class imbalances. A case-control experiment is one of the only ways to take efficient advantage of all minority class observations in a model. The analogous cohort experiment would require identifying a starting alignment date common to all study subjects. Furthermore, longitudinal observational data are often expensive or difficult to acquire, compared to the ease of one-shot, non-temporal case-control datasets. Without the use of retrospective observations, a case-control study is one of the only types that can be conducted immediately after the study is conceived, rather than waiting for observations to be generated, as in prospective studies.

More concerningly, publication biases towards larger effect sizes and higher accuracy may have driven researchers towards methods that accentuate the differences between cases and controls. Temporal bias can be interpreted as a relatively invisible symptom of this subconscious aversion towards ambiguity in prognostic models. Strong predictive models (in terms of accuracy) are naturally easier to create when structural differences between the two groups are used to provide additional signal. The increasing popularity of large data sets and difficult-to-interpret deep learning techniques facilitates this strategy.

This is not to say that case-control studies should be abandoned wholesale. These studies for practical reasons (data efficiency, cost, ease of deployment) have contributed countless numbers of discoveries across fields. However, a systematic understanding of where and why temporal bias exists is critical in the transition of research findings to applications in the clinic and beyond. There are several strategies to minimize temporal bias where it exists and evaluate its effects otherwise (Fig. 4C–E, examples are provided in Supplementary Note 2).

1. Assuming that a suitable control population can be identified, the following two conditions can enable uniform sampling of the control-to-case trajectory: i) the use of a randomized lookback time, and ii) the length of the maximum lookback time plus the length of the observation window is longer than the transition period.
2. Person-time classification or prediction tasks, where multiple windows are drawn from sufficiently extended case observations for use can also uniformly sample the trajectory in question. This approach takes the form of sampling case trajectories more than once, and weighing them according to prevalence. This can be facilitated through careful control criteria definitions, as the selection of sicker controls can shorten the trajectory considered in the experiment, likely at the cost of model discriminative ability.
3. The use of well-defined baseline dates in cohort studies can eliminate temporal bias. Assessing exposure after a particular birthday, at the start of a particular month/year, or after a well-defined event makes the prospective deployment population easier to identify.

Finally, sensitivity analyses combined with researchers’ background domain knowledge regarding the state transition trajectory in question can be used to estimate effects of prospective deployment. An increasing focus on considering the deployability of a given model, the nature of the underlying trajectory, or even whether a particular feature can realistically be predicted from features at hand can also serve to prevent temporal bias from infiltrating a study.

While temporal bias is common and has far reaching implications, it is unique among experimental or epistemological flaws in that once understood, it is fairly easy to detect. As experiments grow broader in scope, transparency regarding the extent to which temporal bias influences findings is key to ensuring the consistency of associations and predictions.

## Methods

### Lipoprotein(a) trajectory imputation

Centiles of lipoprotein A values [Lp(a)] for myocardial infarction (MI) of 4441 Chinese patients (cases) and healthy matched controls (controls) published by Paré et al.^27^ were used to construct log-normal distributions of Lp(a) values for each cohort. One hundred fifty thousand case and control measurements were drawn and a linear model was fit to establish the baseline coefficient of association between Lp(a) and MI in the presence of temporal bias. For trajectory imputation, for each case patient, a starting Lp(a) value was generated using one of three methods: (i) random sampling from the control distribution such that the drawn value is smaller than the case value, (ii) percentile matching (if the case value fell in the Nth percentile of the case distribution, the Nth percentile value from the control was drawn), and (iii) a uniform shift of 15% (representing the observation that the median control value was 15% lower than the median case). This starting value is understood to represent the Lp(a) measurement of the case patient in the distant past at the point when they were cardiovascularly healthy. The case-ending value was directly drawn from the published distributions. For each pair of case-starting and case-ending values, a linear/logarithmic/logistic/step function was fit using the two values as starting and ending points. New case observations were generated by randomly selecting a point along the generated trajectory allowing for the computation of a prospective effect size. All individual experiments were repeated 100 times with newly drawn sample cohorts.

To examine the potential impact of inadvertent selection bias on the observed association between Lp(a) and MI, the Lp(a) values and MI for all patients with more than one Lp(a) observations prior to the first recorded MI event were extracted from the Partners Research Patient Data Registry database in a deidentified manner. This work was approved by the Partners Institutional Review Board (Protocol #2018P000016). Case and control patients were defined based on MI status, and for each patient in each cohort, the (i) largest available, (ii) smallest available, and (iii) mean Lp(a) values were computed and used to identify the observed effect size under each selection scheme by fitting a logistic regression model. All calculations were conducted in R (version 3.44) using the glmnet package, version 2.0-16.

### Delivery prediction from sequential claims data

Records of health insurance claims in 2015 from a deidentified national database from Aetna, a commercial managed health care company, were utilized for this study. The Harvard Medical School Institutional Review Board waived the requirement for patient consent for analysis of this database as it was deemed to not be human subjects research. Delivery events were identified based on International Classification of Diseases (ICD9/10) diagnostic code, Current Procedural Terminology (CPT) code, or the birth year of newly born members linked by subscriber-parent annotations. Cases were defined as individuals who experienced a delivery between February and December, 2015, while controls were defined as individuals who did not experience a delivery during any of 2015. Thirty thousand cases were randomly selected and matched to 30,000 controls based on age and ZIP code. For each individual, case-control and cohort feature windows were defined. Case-control windows were set as the month of records that was three months prior to the delivery/matched baseline date for cases and controls respectively. Cohort windows were set as the month of records from January, 2015. Three studies were simulated: (1) The CC-CC study consisted of model training using case-control windows and model evaluation using case-control windows. (2) The CC-Cohort study consisted of model training using case-control windows and model evaluation using cohort windows. (3) The Cohort-Cohort study consisted of model training using cohort windows and model evaluation using cohort windows. For each study, deep recurrent neural networks and logistic regression models were trained over the features present in each window. For deep recurrent neural network-based models, the linear sequence of features inside the window was provided in the form of International Classification of Diseases (ICD9) codes for diagnoses, Current Procedural Terminology (CPT) codes for procedures, and National Drug Codes (NDC) for prescriptions. The sequence length was set to 20 events, individual sequences were either padded or clipped to meet this requirement. Logistic regression models utilized binary occurrence matrices for all events as features. Both models contained demographic information in the form of age. Sex was excluded as a feature because all cohort members were female. All calculations were conducted in Python 2.7.3 using the Keras 2.2.0 and scikit-learn 0.18.1 packages.

### Simulation of olive oil/myocardial infarction case-control study

Data from the Nurses’ Health Study (NHS) was used for this analysis. All nutrition and disease incidence surveys between 1994 and 2010 were considered. Internal NHS definitions of first MI were utilized to define the case population. Case individuals were only considered if they had at least two consecutive nutritional surveys with answers to all olive oil related questions prior to the first MI event. Individuals with any history of cardiovascular disease including MI and angina were excluded from the control population. Control individuals were only considered if they had at least two consecutive nutritional surveys with answers to all olive oil related questions. In total, 3188 total qualifying MI individuals were identified, and 94,893 controls. A baseline date for each control individual was defined based on the availability of consecutive nutrition surveys. For each case, a matched control was identified based using age at baseline and sex. For all individuals, total cumulative yearly olive oil consumption was computed by summing olive oil added to food and olive oil salad dressing consumption, as validated by Guasch-Ferré et al.^40^. For each experiment, a lookback time between 1 and 4 years was selected, and the cumulative total olive oil consumed during the lookback time relative to the MI date/baseline was calculated. For each lookback time, the effect size between the top quintile (based on total consumption) and the remaining population and statistical significance were calculated using a two-sided *t*-test. Each experiment, including case-control matching, was repeated 200 times. All calculations were conducted in R (version 3.44) using the glmnet package, version 2.0-16.

### Reporting summary

Further information on research design is available in the Nature Research Reporting Summary linked to this article.

## Supplementary information

Supplementary Information

Reporting Summary

## Data Availability

The data that support the findings of this study are available from Aetna Insurance, but restrictions apply to the availability of these data, which were used under license for the current study, and so are not publicly available. Please contact N. Palmer (nathan_palmer@hms.harvard.edu) for inquiries about the Aetna dataset. Summary data are, however, available from the authors upon reasonable request and with permission of Aetna Insurance. All data utilized in the study from the Nurses’ Health Study (NHS) is available upon request with the permission of the NHS and can be accessed at https://www.nurseshealthstudy.org/researchers. All data utilized in the study from the Partners Research Patient Data Registry is available upon request with the permission of Partners Healthcare and can be accessed at https://rc.partners.org/research-apps-and-services/identify-subjects-request-data#research-patient-data-registry.

## Code availability

Auxiliary code is available at https://github.com/william-yuan/temporalbias
