# Supplementary material for: Temporal bias in case-control design: preventing reliable predictions of the future
Source: Nat Commun. 2021 Feb 17;12:1107. doi: 10.1038/s41467-021-21390-2 (PMC7889612; doi:10.1038/s41467-021-21390-2)
Supplement: Supplementary file 1 — Supplementary Information [file 41467_2021_21390_MOESM1_ESM.docx]

Supplementary Information for *Temporal Bias in Case-Control Design: Preventing Reliable Predictions of the Future:* :

Supplementary Table 1: Variables and Descriptions

| Variable | Description |
| --- | --- |
| s | Given an observation in a case individual, the time of the observation relative to the future event. Always a negative value. |
| f(s) | Trajectory of observations and predictors towards the future event. General form:  $f\left( s \right)= \left\{ \begin{aligned} \bar{F}_{C} \mathrm{if} s \leq s_{c} \\ g\left( s \right)\mathrm{if} s>s_{c} \mathrm{where} \lim_{s\to0} g(s)= \bar{F}_{s=0} \end{aligned} \right.$ |
| Case population | Defined by the presence of the event, aligned in time such that events among all individuals happen simultaneously (s=0). |
| Control population | Defined by absence of event. |
| $F_{s=0}$ | Distribution of observed f(s) at s=0. |
| $F_{C}$ | Distribution of observed f(s) of control population. |
| $w_{1}$,$w_{2}$ | Relative weight of control and case populations respectively. |
| $s_{C}$ | Minimum magnitude timegap s when $F_{C}$ and $w_{1}C +w_{2}F_{s=0}$are identically distributed. |
| $F_{s_{C}}$ | Distribution of observed f(s) at s=$s_{C}$. |
| i | Lookback time, defined by the experimenter during a study. A positive value. |
| j | Observation window, defined by the experimenter during a study. A positive value. |
| k | Defined as ${i+j+s}_{C}$ |

Supplementary Note 1: Theoretical Framework for Temporal Bias

We present a theoretical framework for understanding temporal bias for an arbitrary case-control study. These types of studies can have two objectives, 1) to utilize observed features to predict the transition of controls to cases or 2) to evaluate the strength of association between observed features and case-control transition. The case population is defined by the first occurrence of a characteristic event that serves to align population members in time. This event is the subject of the study: individuals who experience the event are cases while those who do not are potential controls. We next define a timegap $s$that represents the amount of time between an observation and the characteristic event. For every member of this population, this event is fixed to occur at timegap $s = 0$. We next define a control (non-case) population whose members are i) representative of the population that produced the cases and ii) are at risk of experiencing the event in question. Let$f(s)$ represent the predictor trajectory (Condition 2, Background). Let $F_{s=0}$ and $F_{C}$ represent the distribution of observed $f(s)$ for the case population at $s=0$and for the control population, respectively. Note that individual observations from $F_{C}$ do not correspond to uniform values $s$ because controls do not have an event against which to index, by definition. We define $s_{C}$ as the minimum timegap when $F_{C}$ and $w_{1}F_{C} +w_{2}F_{s_{C}}$ are identically distributed (Condition 3, Background), where $w_{1}$ and $w_{2}$are the relative populations of the control and case population, respectively, and $F_{s_{C}}$ is the distribution of observed $f(s)$ among cases at $s_{C}$*.* That is, $s_{C}$ is defined as the earliest point where the cases begin to diverge from the controls. Because controls are selected due to not experiencing the event, their observations correspond to otherwise unknown values of $s\leq s_{C}$ (Figure 3B). By definition, controls transition to cases and not vice versa, so $\max(f(s))=F_{s=0}$ and $\min(f(s))=F_{C}$ (Condition 1, Background). Finally, we note that $F_{C}$ does not necessarily represent zero progression; it is, rather, an abstraction of the baseline progression of the control population toward the event in question. This is a representation of the fact that there is a non-zero chance that controls transition to cases, assuming controls were properly selected with exchangeability in mind. We can now express the general form of $f(s)$*:* for $s\leq s_{C}$, $f(s)=F_{C}$; for $s>s_{C}$*,* $f(s)$ increases to $F_{s=0}$ at $s=0$. This process appears to introduce a discontinuity in $f(s)$ when $s=s_{C}$. This is an artifact of the binary nature of the case-control experiment. In essence, although the ground truth trajectory may be continuous, the experiment naturally uses the observed control distribution to define a cutoff beneath which controls are considered exchangeable. The discontinuity occurs when the true trajectory intersects with this cutoff.

During the experiment itself, a look-back time $i$ and observation window $j$ are defined where $i,j\geq0$. For static time point predictions, $j$ = 0. These values are not necessarily constant for all features or across all patients. Observations of the case population correspond to those made at values of $s$ between $\left[ -i-j, -i \right]$, while observations of the control population still correspond to $s \leq s_{C}$ (where all observations are members of the $F_{C}$ distribution). When $i=j=0$, the case observations correspond directly to $s=0$. We assume that $i$ is chosen by the experimenter based on the period of time when a prediction or evaluation would be useful. Therefore, in prospective deployment, individuals with observations made at $s > -i$ will not need to be considered for the purpose of model evaluation. Comparisons between the case observations ${[F}_{-i-j},F_{-i}]$ and control populations $F_{C}$can then be made, leading to odds ratios for observations and the event, or predictive models for the event.

Temporal bias occurs when $i+j<{|s}_{C}|$ and $\frac{1}{j}\int_{-i-j}^{-i} f(s) ds>\frac{1}{k}\int_{s_{C}}^{-i-j} f(s) ds$, that is, when the average value of $f\left( s \right)$ in the red region (Supplementary Figure 1, observed by the experimenter) is higher than the average value of $f\left( s \right)$ in the white region (Supplementary Figure 1, unobserved by the experimenter). while the magnitude of the bias can be estimated by $\frac{\int_{s_{C}}^{-i} f(s) ds}{f\left( -i \right)*({k+j+s}_{C})}$, that is, the ratio between the prospectively observed trajectory (the value of $f\left( s \right)$ evaluated from ${s =s}_{C}$ to $s = -i$*)* and the experimentally observed risk (the highest value of $f\left( s \right)$ within the red region, between the same interval). Prospective deployment of an odds ratio or model can be thought of as randomly sampling $f\left( s \right)$ over s between $\left[ s_{C}, 0 \right]$. Note that this task is impossible: all values of $s$ are negative and defined relative to a case event occuring in the future. The precise value of $s$ for a given individual cannot be known until this event happens, while if an individual is censored, this value will never be known. The tension of using $s$, rather than conventional time, is an artifact of the fact that case-control studies intrinsically leak information regarding case status from the future. The case-control window structure assumes an instantaneous transition between $s=0$ and $s= s_{c}+i+j$. As we show, this results in an exaggeration of odds ratios or predictive accuracies due to sampling cases artificially at timepoints close to the event.


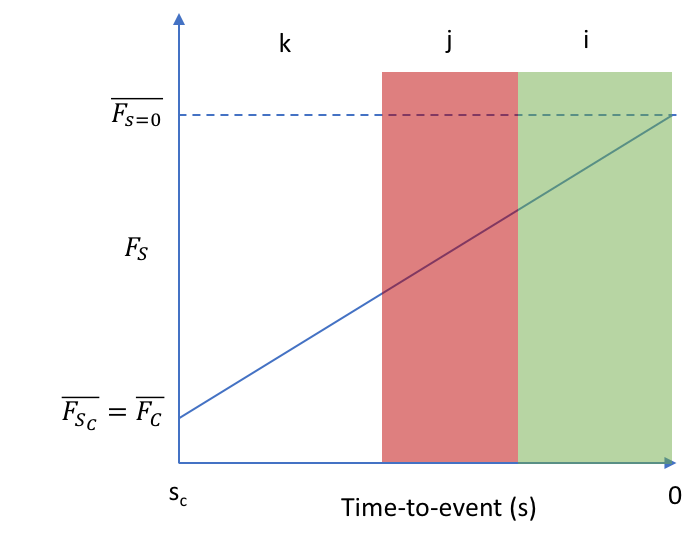


Supplementary Figure 1: Schematic of Generic Temporal Bias Scheme. The investigator attempts to use features in the observation window (j) with a prediction window offset (i) to predict an individual’s position across the entire trajectory: an impossible task.

Supplementary Note 2:

Examples of Case-Control Studies Immune to Temporal Bias

- Biobank studies can consist of enrollment periods where individuals are extensively phenotyped at what can be thought of as a random point in time relative to any underlying trajectories, and have follow-up mechanisms regarding potential outcomes. Consider a case-control study that compared the nutritional profiles of biobank participants who experienced an MI to the profiles of participants who did not. Because the timepoint considered is not indexed to the date of the MI, the study should be immune to temporal bias, so long as cardiovascular health was not a condition of recruitment (Figure 4C).
- Embedding case-control studies within cohorts can fortify them against temporal bias. Consider the olive-oil consumption/MI studies simulated using the Nurses’ Health Study dataset. If, for each individual, random timepoints were selected for olive-oil consumption evaluation, rather than points within a well-defined time of the MI, the overall MI trajectory would be more evenly sampled (Figure 4C).
- The onset of sepsis among ICU patients is a common task for prediction. Because patients are monitored over time, models that evaluate sepsis risk over every available timepoint for every case can be said to uniformly sample the trajectory of ICU admission → sepsis (Figure 4D)
