# Supplementary material for: Temporal bias in case-control design: preventing reliable predictions of the future
Source: Nat Commun. 2021 Feb 17;12:1107. doi: 10.1038/s41467-021-21390-2 (PMC7889612; doi:10.1038/s41467-021-21390-2)
Supplement: Supplementary file 2 — Reporting Summary [file 41467_2021_21390_MOESM2_ESM.pdf]

nature research

Corresponding author(s): William Yuan

Last updated by author(s): Jan 12, 2021

Reporting Summary

Nature Research wishes to improve the reproducibility of the work that we publish. This form provides structure for consistency and transparency in reporting. For further information on Nature Research policies, see our Editorial Policies and the Editorial Policy Checklist.

Statistics

For all statistical analyses, confirm that the following items are present in the figure legend, table legend, main text, or Methods section.

n/a	Confirmed	
☐	☒	The exact sample size (n) for each experimental group/condition, given as a discrete number and unit of measurement
☒	☐	A statement on whether measurements were taken from distinct samples or whether the same sample was measured repeatedly
☐	☒	The statistical test(s) used AND whether they are one- or two-sided<br>*Only common tests should be described solely by name; describe more complex techniques in the Methods section.*
☐	☒	A description of all covariates tested
☐	☒	A description of any assumptions or corrections, such as tests of normality and adjustment for multiple comparisons
☐	☒	A full description of the statistical parameters including central tendency (e.g. means) or other basic estimates (e.g. regression coefficient) AND variation (e.g. standard deviation) or associated estimates of uncertainty (e.g. confidence intervals)
☐	☒	For null hypothesis testing, the test statistic (e.g. F, t, r) with confidence intervals, effect sizes, degrees of freedom and P value noted<br>*Give P values as exact values whenever suitable.*
☒	☐	For Bayesian analysis, information on the choice of priors and Markov chain Monte Carlo settings
☒	☐	For hierarchical and complex designs, identification of the appropriate level for tests and full reporting of outcomes
☐	☒	Estimates of effect sizes (e.g. Cohen's d, Pearson's r), indicating how they were calculated

Our web collection on statistics for biologists contains articles on many of the points above.

Software and code

Policy information about availability of computer code

Data collection	No software was used.
Data analysis	Custom R and Python code was utilized for data analysis and has been attached as auxillary files.

For manuscripts utilizing custom algorithms or software that are central to the research but not yet described in published literature, software must be made available to editors and reviewers. We strongly encourage code deposition in a community repository (e.g. GitHub). See the Nature Research guidelines for submitting code & software for further information.

Data

Policy information about availability of data

All manuscripts must include a data availability statement. This statement should provide the following information, where applicable:

- Accession codes, unique identifiers, or web links for publicly available datasets
- A list of figures that have associated raw data
- A description of any restrictions on data availability

All data utilized in the study from Aetna Inc. is available upon request with the permission of Aetna Inc. All data utilized in the study from Partners Healthcare is available upon request with the permission of Partners Healthcare. All data utilized in the study from the Nurses' Health Study (NHS) is available upon request with the permission of the NHS. All data utilized in the study from the Partners Research Patient Data Registry is available upon request with the permission of Partners Healthcare.

Field-specific reporting

Please select the one below that is the best fit for your research. If you are not sure, read the appropriate sections before making your selection.

- [x] Life sciences
- [ ] Behavioural & social sciences
- [ ] Ecological, evolutionary & environmental sciences

For a reference copy of the document with all sections, see nature.com/documents/nr-reporting-summary-flat.pdf

Life sciences study design

All studies must disclose on these points even when the disclosure is negative.

Sample size	Sample sizes were chosen according to data availability and computer hardware constraints. However, in each analysis, sample sizes were significantly larger than literature references.<br>Trajectory imputation: The Pare study utilized records of 12000 patients, stratified by ethnicity, of which, the largest subgroup, Chinese, had 4500 patients. Our simulations consisted of 150,000 patients per replication to ensure timely completion of simulations.<br>Delivery prediction: This study was not modeled after any particular literature study. We utilized a balanced set of 60,000 patients. Papers attempting similar prediction tasks (Choi et al), utilized 32000 patients for a significantly more difficult outcome (heart failure) and a less balanced cohort of cases/controls (1 to 8).<br>Olive oil: we utilized a set of 3200 cases and 95000 controls. The Bertuzzi and Fernandez-Jarne studies used less than 2000 patients combined.
Data exclusions	Data was only excluded in the case of the olive oil analysis. Individuals with any history of cardiovascular disease including MI and angina were excluded from the control population, matching the inclusion criteria of the Bertuzzi and Fernandez-Jarne studies. Control individuals were only considered if they had at least two consecutive nutritional surveys with answers to all olive oil related questions. Case individuals were only considered if they had at least two consecutive nutritional surveys with answers to all olive oil related questions prior to the first MI event. These criteria are required to examine the time-dependence of olive oil consumption.
Replication	Trajectory imputation: experiments were repeated 100 times with newly drawn cohorts<br>Delivery Prediction: models were retrained 10 times each using novel cohorts to produce confidence intervals on AUC estimates.<br>Olive Oil study: overall findings cannot be replicated, but individual experiments were replicated 200 times for each year-lookback period.<br>All attempts were successful.
Randomization	Randomization was not relevant to this study as all analysis was retrospective.
Blinding	Blinding was not relevant to this study as all analysis was retrospective.

Reporting for specific materials, systems and methods

We require information from authors about some types of materials, experimental systems and methods used in many studies. Here, indicate whether each material, system or method listed is relevant to your study. If you are not sure if a list item applies to your research, read the appropriate section before selecting a response.

Materials & experimental systems

n/a	Involved in the study	
☒	☐	Antibodies
☒	☐	Eukaryotic cell lines
☒	☐	Palaeontology and archaeology
☒	☐	Animals and other organisms
☒	☐	Human research participants
☒	☐	Clinical data
☒	☐	Dual use research of concern

Methods

n/a	Involved in the study	
☒	☐	ChIP-seq
☒	☐	Flow cytometry
☒	☐	MRI-based neuroimaging
